# Challenges and solutions to nicotine replacement therapy access: observations from SCIMITAR+

**DOI:** 10.1192/bjo.2020.100

**Published:** 2020-10-15

**Authors:** Catherine E. Arundel, Emily Peckham, Della Bailey, Suzanne Crosland, Paul Heron, Simon Gilbody

**Affiliations:** Department of Health Sciences, University of York, UK; Department of Health Sciences, University of York, UK; Department of Health Sciences, University of York, UK; Department of Health Sciences, University of York, UK; Department of Health Sciences, University of York, UK; Department of Health Sciences, University of York, UK

**Keywords:** Smoking cessation, nicotine replacement therapy, smoking cessation services, severe mental ill health, barriers and facilitators

## Abstract

**Background:**

Given that smoking results in poor physical and mental health, reducing tobacco harm is of high importance. Recommendations published by the National Institute for Health and Care Excellence to reduce smoking harms included provision of support, use of nicotine containing products and commissioning of smoking cessation services.

**Aims:**

This report explores the difficulties in obtaining such support, as observed in a recently conducted randomised controlled trial in patients with severe mental ill health, and outlines suggestions to improve facilitation of provision.

**Method:**

Data collected during the Smoking Cessation Intervention for Severe Mental Ill Health Trial (SCIMITAR+) (trial Registration ISRCTN72955454), was reviewed to identify the difficulties experienced, across the trial, with regards to access and provision of nicotine replacements therapy (NRT). Actions taken to facilitate access and provision of NRT were collated to outline how provision could be better facilitated.

**Results:**

Access to NRT varied across study settings and in some instances proved impossible for patients to access. Difficulty in access was irrespective of a diagnosis of severe mental ill health. Where NRT was provided, this was not always provided in accordance with NICE guidelines.

**Conclusions:**

Availability of smoking cessation support, and NRT provision would benefit from being made clearer, simpler and more easily accessible so as to enhance smoking cessation rates.

## Background

Figures suggest that 57–68 % of people with severe mental ill health (SMI) smoke tobacco compared with 19 % in the general population,^1,2^ resulting in an average loss of 17 years of life on account of smoking.^2^ Reducing tobacco harm in people with SMI is therefore of high importance.

The National Institute for Health and Care Excellence (NICE) guidance (PH48),^3^ focused on reducing smoking harms in secondary care and mental health settings through provision of support, use of nicotine containing products and commissioning of smoking cessation services. Both NICE PH48^3^ and guidance from the Royal College of General Practitioners and Royal College of Psychiatrists^4^ propose that people with SMI should be offered behavioural support, in addition to two forms of nicotine replacement therapy (NRT), given that this increases the odds of smoking cessation with little adverse effect.^3–5^

Despite this guidance, smoking cessation service spending reduced by £41 million between 2014/2015 and 2017/2018,^6^ and a 75 % reduction in prescribed smoking cessation products has been identified.^7^ Given the impact smoking has on overall health and that this is the single most modifiable risk factor for early death, this is of significant concern.

## Aims

The recent Smoking Cessation Intervention for Severe Mental Ill Health Trial (SCIMITAR+, trial registration: ISRCTN72955454),^8^ recruited participants between 7 October 2015 and 16 December 2016 to evaluate the effectiveness of a bespoke smoking cessation intervention using tailored support compared with regular smoking cessation provision for people with SMI.^8^ As part of the intervention, participants were encouraged to use two forms of NRT, in accordance with those recommendations as detailed above.^3,4^

During the trial,^8^ access to NRT varied across study settings and in some instances proved impossible for patients to obtain. This brief report describes the differences and difficulties in NRT access, in the context of people with SMI, and aims to identify how NRT access could be better facilitated, both for those with SMI and also in the general population.

## Method

Data collected during SCIMITAR+, was reviewed to identify the difficulties experienced, across the trial with regards to access and provision of NRT. Actions taken to facilitate access and provision of NRT were collated.

### Ethical approval and consent to participate

The authors assert that all procedures contributing to this work comply with the ethical standards of the relevant national and institutional committees on human experimentation and with the Helsinki Declaration of 1975, as revised in 2008. This work was conducted as part of SCIMITAR+ for which ethical approval was obtained: NRES Committee Yorkshire and The Humber – Leeds East Research Ethics Committee on 19 March 2015 (ref: 15/YH/0051261). SCIMITAR+ obtained written informed consent from all participants.

## Results

There were a number of barriers to NRT access.

### Barriers to NRT access

Initially in SCIMITAR+, smoking cessation services were provided by general practitioner (GP) practices. As the study progressed, smoking cessation provision diversified with provision being supplied by GP practices, the local government Stop Smoking Services, charitable Stop Smoking Services and hospitals, either independently or in collaboration with other groups. While NRT is available to purchase, for low income groups cost was a barrier and so NRT was usually provided at a lower cost directly by the local service providing smoking cessation support, or via the GP through prescription.

In some areas of the UK, NRT prescribing had been devolved from GP practices to the local Stop Smoking Services run by the local government or charities. Where this had occurred, the change was not always clearly publicised and so it was often difficult to identify who was responsible for local prescribing. Having to navigate complicated local arrangements to obtain support, and NRT, has the potential to disincentivise patients from pursuing a smoking cessation attempt.

Where GP practices remained responsible for NRT prescribing, rarely were prescription requests rejected. Where these were rejected, this was especially pertinent where patients had expressed an interest in using alternative evidence-based pharmacological treatments (for example varenicline) as some GPs deemed that these products were not appropriate for use in patients with SMI. This is likely the result of earlier reports^9^ that suggested use of these medications may increase psychiatric symptoms or events in patients with SMI, however, this has subsequently been refuted.^10^ In some cases, prescribing by the mental health team was required to facilitate patient access to NRT; however, this was not a sustainable alternative to a GP prescribing in the long term.

Where funding for smoking cessation services was devolved to local government, but was not protected, this resulted in a reduction in smoking cessation services, which were only available to specific patient groups for example, pregnant women and patients with respiratory disorders.

Where access was available, the levels of prescribing were restricted either to one type of NRT or for a defined period of prescribing only, largely because of services trying to preserve limited available funding. This contravenes the recommendations made both in terms of prescribing quantity and duration^4^ and may result in patients being either disincentivised to, or to be able to sustain a quit attempt.

Increasing prescription costs were also identified as a barrier for some patients, particularly those who were not in receipt of prescription fee exemption (for example those not in receipt of welfare benefit). The most significant impact of this was on initial prescribing ahead of smoking cessation or on patients cutting down prior to making a quit attempt (cutting down to quit). As the patient decreased or ceased their cigarette use, the savings made will likely have off-set this financial burden to some degree.

## Discussion

Despite the recommendations made in both NICE and UK Primary Care Guidance documents,^3,4^ SCIMITAR+ identified distinct variability in provision of NRT for patients wishing to quit smoking.

### Implications

Given the impact smoking has on overall health and that this is the single most modifiable risk factor for early death, reversing the decline in access is imperative to ensure patients with a current history of smoking are given sufficient resources to make, and to sustain, a quit attempt. In line with previous guidance,^3,4^ it is suggested that resource is reviewed and increased, so as to facilitate ease of access to relevant support services and medications. Education among healthcare practitioners is also important to dispel misconceptions regarding use of smoking cessation medication and to increase awareness of clinical prescribing guidelines.

Within SCIMITAR+, it was possible to remove or reduce some of the barriers through provision of centralised support to enable access to NRT, coordinated and delivered by a member of the trial team. This contact enabled misconceptions regarding NRT use or smoking cessation in this population to be tackled and concerns to be allayed, and resolution methods identified and implemented on a case by case basis.

As a result, a single, centralised contact with services providing NRT may be useful to facilitate education around and coordination of NRT provision in a standardised manner across the UK. Where the responsibility of smoking cessation provision has been devolved to local governments, it is also suggested that funding is protected to enable appropriate provision to be provided to those who require access to support and NRT provision.

In the context of further research in relation to smoking cessation, it is suggested that study teams should consider, from the outset, how best to facilitate NRT provision from smoking cessation services. It may be relevant to obtain approvals to provide NRT within the trial setting; however, this may limit the transferability of effective strategies into routine practice, if NRT access continues to be restricted and/or is difficult. Where NRT is to be provided through routine prescribing, it is suggested that study teams consider implementing a centralised point of support within the trial team to facilitate this.

In conclusion, evaluation of activity and experiences observed in the conduct of SCIMITAR+ has identified that NRT is routinely difficult for patients to access. Where access is available, decommissioning or service cuts mean that two forms of NRT are not always provided, and provision of products is for a fixed-time period only. Availability of smoking cessation support, and NRT provision would benefit from being made clearer, simpler and more easily accessible so as to enhance smoking cessation rates.

## Data Availability

Data availability is not applicable to this article as no new data were created or analysed in this study.
